# Clinical and radiographic evaluation for two crestal sinus lift techniques: osteotome versus osseodensification. a systematic review and meta-analysis

**DOI:** 10.1186/s40729-025-00615-9

**Published:** 2025-05-16

**Authors:** Carlos Manuel Cobo-Vázquez, Sonia García-Rodríguez, María Eugenia Colmenares-Otero, Luis Miguel Sáez-Alcaide, Jorge Cortés-Bretón-Brinkmann, Cristina Madrigal Martínez-Pereda, Cristina Meniz-Garcia

**Affiliations:** Department of Clinical Specialities. Faculty of Dentistry, Complutense University of Madrid, Plaza Ramón y Cajal, 3, 28040 Madrid, Spain

**Keywords:** Maxillary sinus lift, Crestal sinus lift, Osseodensification, Osteotome

## Abstract

**Purpose:**

Maxillary sinus floor elevation is a safe and effective surgical technique for achieving vertical bone height, performed through either a lateral or crestal approach. The latter includes both the osteotome technique and osseodensification. The aim of this systematic review was to compare the outcomes of the classic crestal sinus lift technique and the osseodensification sinus lift approach in terms of the bone gain, marginal bone loss, survival rate, follow-up time and complications.

**Methods:**

This review was performed following PRISMA guidelines. An electronic search was conducted across three databases: (1) The National Library of Medicine (MEDLINE/PubMed); (2) SCOPUS; and (3) Cochrane Central Register of Controlled Trials (CENTRAL). The Newcastle–Ottawa Quality Assessment Scale and the Cochrane Collaboration tool for evaluating risk of bias. A meta-analysis for random effects was carried out for implant survival, residual bone height and bone gain.

**Results:**

Thirteen studies were included, ten studies performed the osteotome (OST) approach and three performed the osseodensification (OD) approach, with a total of 519 sites treated. The residual bone height was 5.94 and 5.00 mm for OD and OST, respectively. For bone gain, similar results were found for both groups, being 3.37 mm for OD and 3.18 mm for OST. For both groups, the most used diameter and length of the implant was 4 and 10 mm, respectively, and the implant survival rates ranged from 94.1% to 100%. OST technique reflected a complication rate of 14.32%, compared to the OD technique, which showed a complication rate of 2.78%.

**Conclusions:**

It can be concluded that the maxillary sinus lift by osseodesinfication approach is a safe, predictable and successful technique compared to the osteotome approach, with similar outcomes regarding bone gain which is an important parameter for implant placement.

## Background

Edentulism is a highly prevalent condition on a global scale [1]. Moreover, dental loss has a detrimental impact on the quality of life [2] with repercussions on oral function and related with low self-esteem [3]. Nowadays, the therapeutic value of dental implants has accomplished the gold standard among restorative alternatives for missing teeth, although the possible risk of biological and technical complications has to be assets [4, 5]. However, the implant placement in the upper jaw is not only challenging in some cases due to the loss of alveolar ridge dimensions that occurs physiologically when a tooth is lost but also as a result of potential sinus pneumatization [6].

Additionally, the bone density in the maxillar is generally low, presenting an extra challenge for successful implant placement. This complex remodeling, along with the presence of the maxillary sinus and a reduction in alveolar bone height, can influence treatment planning due to the limited bone available for implant placement and potential complications during rehabilitation [6, 8].

Nevertheless, maxillary sinus floor elevation is a safe and an effective surgical technique to gain bone height vertically, performed either via a lateral or crestal approach [7]. There are several methods for sinus lift [8] such as classical technique, using lateral approach with the risk of bone dehiscences [9]; Summers introduced a sinus augmentation technique that utilized a crestal approach with a drill and a calibrated osteotome (OST) [10]. Osseodensification (OD) developed by Huwais and Meyer in 2013, uses specialized burs to compact bone and expand the osteotomy, resulting in increased bone density [11, 12].

Other authors have previously described the bone gain dimension accomplished with maxillary lift approach through osteotome and osseodensification [13–15]. However, in this review the bone gain of these surgical techniques is analyzed in conjunction with other variables that provide a more complete view of the safety and success of these techniques.

The aim of this meta-analysis was to compare the outcomes of the osteotome sinus lift technique and the osseodensification sinus lift approach in terms of the survival rate, bone gain, marginal bone loss and complications.

## Materials and methods

### Review development and PICO(s) question

Following the Preferred Reporting Items for Systematic Reviews and Meta-Analysis Protocols (PRISMA-P) recommendations [16], the ensuing PICO (population, intervention, comparison, outcome) model was defined:

- Population: patients aged more than or equal to nineteen years who require maxillary sinus lift and simultaneously implant placement.

- Intervention: crestal maxillary sinus lift by osseodensification approach.

- Comparison: crestal maxillary sinus lift by osteotomes approach.

- Outcomes: the main variables studied provide clinical and radiographic related to residual bone height, bone gain, implant's survival and marginal bone loss in addition to intra- and post-operative complications of maxillary sinus lift either through osseodensification or osteotome approach. Secondary variables included: implant size, implant loading and follow-up time.

- Study design: randomized controlled trials, observational studies (prospective and retrospective studies), cross-sectional studies, case-control studies issued in scientific journals.

As stated by the PICOS framework, [16] the review question established was "Is the maxillary sinus lift (population) by osseodesinfication approach (intervention) a safe, predictable and successful technique (outcome) compared to the osteotome approach (comparison)?".

### Eligibility criteria

The inclusion criteria were as follows: Randomized controlled trials, prospective and retrospective observational studies, cross-sectional studies issued in scientific journals; studies carried out in adult healthy humans; studies executing crestal maxillary sinus lift intervention, either through osseodesinfication or by osteotome approach, in combination with simultaneous implant placement, sample size of at least five patients; follow-up carried out after the surgical intervention ≥ 4 months; studies providing clinical and radiographic parameters regarding residual bone height, bone gain, implant size, implant loading, marginal bone loss, survival rate, follow-up time in addition to intra- and post-operative complications; publication date between 2014 and 2024 and studies written in English or Spanish.

The exclusion criteria were as follows: Bibliographic and systematic reviews, case report, letters to the editor, in vitro and animal studies; studies with short follow-up periods, < 4 months; studies utilizing lateral maxillary sinus lift approach; studies performing sinus lift alone without implant placement; studies using any type of graft for the sinus lift; studies for which the full text was not available; and studies written in a language other than English or Spanish.

### Search strategy

An electronic literature search was conducted for studies published up to October 15th 2024 in three databases: (1) The National Library of Medicine (MEDLINE/PubMed); (2) SCOPUS; and (3) Cochrane Central Register of Controlled Trials (CENTRAL). The search strategy as follows (adapted to each data-base): (((“ossedensification”[All Fields] AND (“paranasal sinuses” [MeSH Terms] AND (“lifting” [MeSH Terms])) OR (“maxillary sinus” [MeSH Terms] OR ((“drug implants” [MeSH Terms]) OR ((“paranasal sinuses” [MeSH Terms] OR “floors and floorcoverings” [MeSH Terms] OR “lifting” [All Fields] OR “lift” [All Fields])) OR (“transcrestal” [All Fields] AND (“implant” [All Fields] OR “implantation” [All Fields] OR “implantitis” [All Fields] AND “osteotome” [All Fields] OR “osteotomic"[All Fields] AND “elevation"[All Fields] OR (“crestal”[All Fields] AND “augmentation"[All Fields] OR “augmented”[All Fields] AND (“Densah” [All Fields] AND “Burs” [All Fields]) AND (“graftless”[All Fields] AND “elevation”[All Fields] OR “elevational"[All Fields] OR “elevations"[All Fields] AND ((clinicaltrial[Filter] OR clinicaltrialprotocol[Filter] OR controlledclinicaltrial[Filter] OR multicenterstudy[Filter] OR randomizedcontrolledtrial[Filter]) AND (humans[Filter]) AND (english[Filter] OR spanish[Filter]) AND (alladult[Filter]) AND (2014:2024[pdat])). To conduct the screening process, all reference were imported into EndNote X9 library.

### Data collection

The search was carried out in the mentioned databases by two reviewers (MEC and SG). A list of the results obtained after eliminating duplicates and triplicates was made, which were approached in the following way: in the first phase, both reviewers (MEC and SG) independently review the titles and abstracts of the studies. The studies shortlisted in this phase were compared by both reviewers. Then, the final selection of the studies was made by reading the articles in full. This phase was carried independently by both reviewers (MEC and SG) to assess the relevance of each study. Any disagreements in the selection process were resolved by discussion with a third reviewer (LMSA).

The data from each included study was collected by the reviewers (MEC and SG) and entered into Google Spreadsheet tables (web version), including the following data: authors, year of publication, study design, number of patients, number of sites (sites where the intervention was performed), type of maxillary sinus augmentation (technique), residual bone height (mm), bone gain (mm), implant size, implant loading, marginal bone loss, survival rate, follow-up and complications.

### Risk of bias in individual studies

The quality of the studies was assessed using the Newcastle–Ottawa Scale (NOS) for cohort and observational studies [17]. This scale evaluates three main categories: the selection of study groups, the comparability between participants, and exposure to the variable under study. Each individual study can achieve a maximum score of nine points. The Critical Appraisal Checklist from the Cochrane Collaboration tool for evaluating risk of bias [18] was employed to assess the quality of Randomized controlled Trials (RCTs). The studies were categorized as follows: low risk of bias (low risk across all key domains), unclear risk of bias (uncertain risk in one or more key domains), and high risk of bias (high risk in one or more key domains).

The systematic review protocol was registered in PROSPERO with number CRD42025623242.

### Statistical analysis

A meta-analysis for random effects was carried out for implant survival, residual bone height and bone gain. A test of homogeneity and heterogeneity with Q statistics and I-squared was done for both groups. A statistically difference significance was assessed as p < 0.005. Meta-analysis quality was evaluated by Egger´s regression-based test. A significance of p < 0.001 was established.

## Results

### Inter-reviewer agreement

The Kappa statics for inter-reviewer agreement between the two independent reviewers (SG and MEC) was 0.95 (98.58% CI: 0.91/ 0.99) for title and abstract selection, and 1 (100% CI: 1/1) for the assessment of full-text inclusion/exclusion. Thus, inter-reviewer agreement was nearly perfect, eliminating the need for a third reviewer for discrepancies.

### Study selection (Fig. 1)

**Fig. 1:**
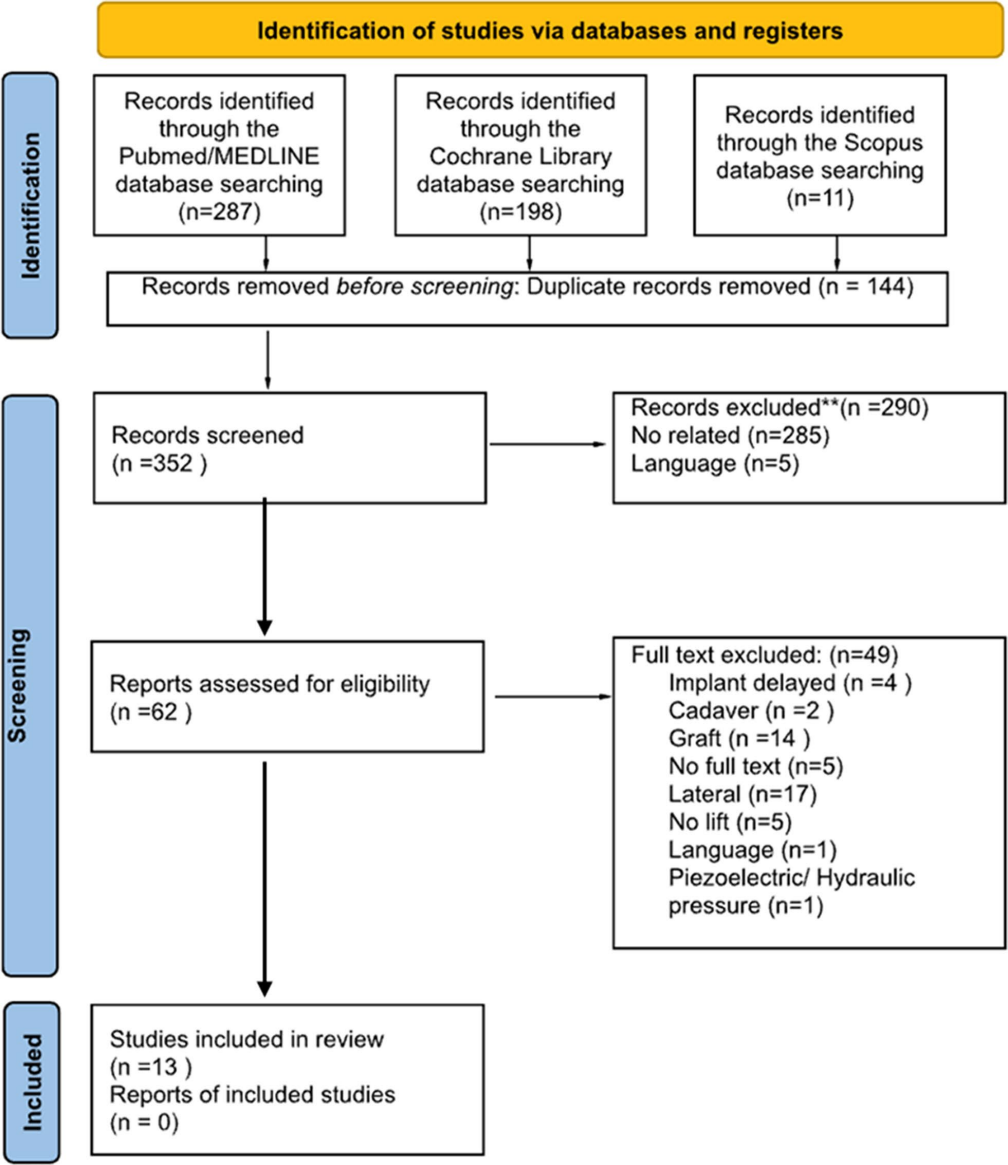
PRISMA flowchart diagram showing selection process

The initial search across the three databases yielded a total of 496 articles, which was reduced to 352 after removing duplicates. Screening of titles and abstracts led to the exclusion of 290 articles: 285 were unrelated to crestal sinus lift techniques (osteotome versus osseodensification), and 5 were not published in English. After a full-text review of the remaining 62 articles, 49 were excluded for the following reasons: studies that placed delayed implants (n = 4) with the lateral sinus lift (n = 17) or no lift (n = 5); studies conducted in cadaver (n = 2); use of bone grafts (n = 14); use of other techniques such as piezoelectric or hydraulic pressure (n = 1). Other studies were excluded because there is no full text available (n = 5).

The PRISMA flowchart (Fig. 1) illustrates the process of searching and selecting articles. This systematic review included 13 full-text articles: 5 Randomized Controlled Clinical Trial, 1 prospective cohort, 1 prospective study, 2 retrospective studies, 3 prospective clinical trial, 1 pilot randomized trial.

### Study characteristics (Table 1)

**Table 1:**
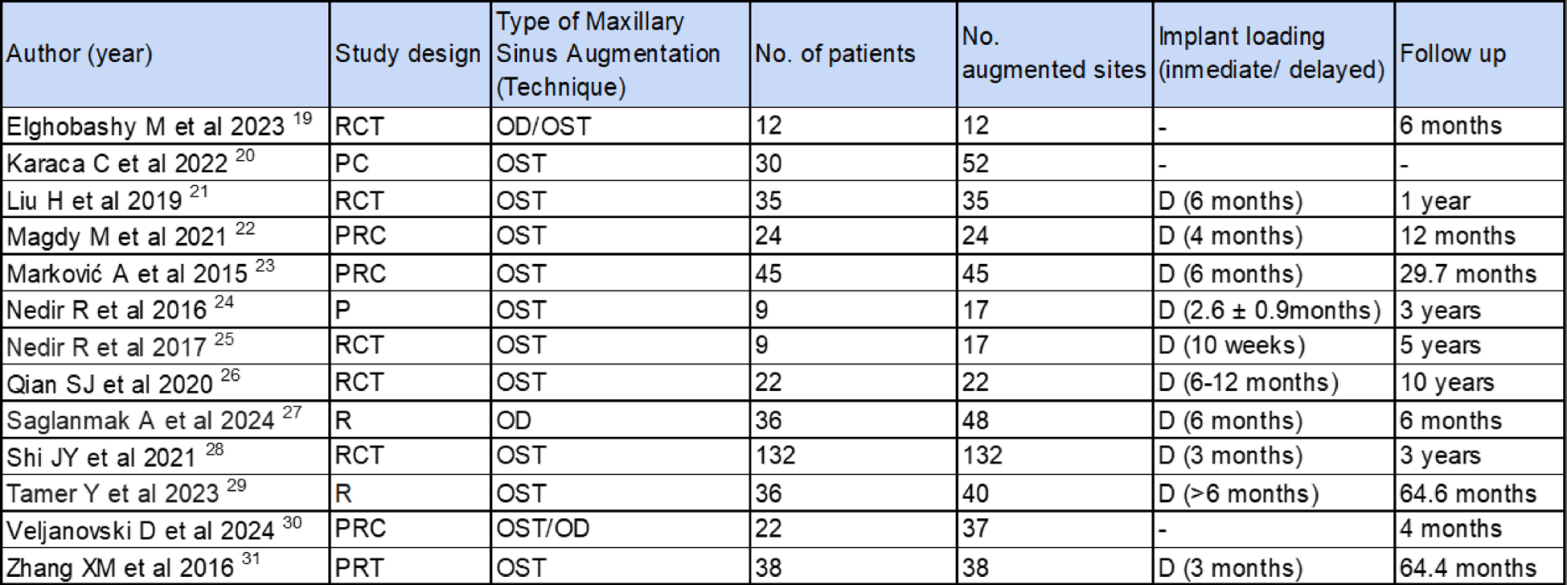
Characteristics for included studies in the systematic review

*RCT* Randomized Clinical Trial, *PC* Prospective Cohort, *P* Prospective, *R* Retrospective, *PRC* Prospective Randomized Clinical, *PRT* Pilot Randomized Clinical, *OD* osseodensification, *OST* osteotome, *D* Delayed

Out of the 13 studies included in the review, 10 used osteotomes for crestal augmentation and 3 employed osseodensification techniques for sinus lift procedures. The studies collectively involved 450 patients with 519 sites treated using crestal approach. The review included only sites treated with osteotomes or osseodensification techniques. Sites treated with lateral sinus lift approach were excluded.

In the included studies, some examine both techniques—osseodensification and osteotome—in parallel, with a total of 34 patients and specifically 49 sites [19, 30]. Other studies evaluate only one of the two techniques [20–29, 31]. For the osteotome technique, a sample of 433 patients was assessed across 494 sites [20–29, 31], while for osseodensification, a sample of 17 patients was studied at 25 sites. [19, 30]

The follow-up period ranged from 4 months to a maximum of 10 years. In both the osteotome and osseodensification techniques, implant loading was deferred in 394 patients an average of 5.3 months, with a minimum of 3 months and a maximum of 12 months (Table 1).

## Results of individual studies (Table 2)

**Table 2:**
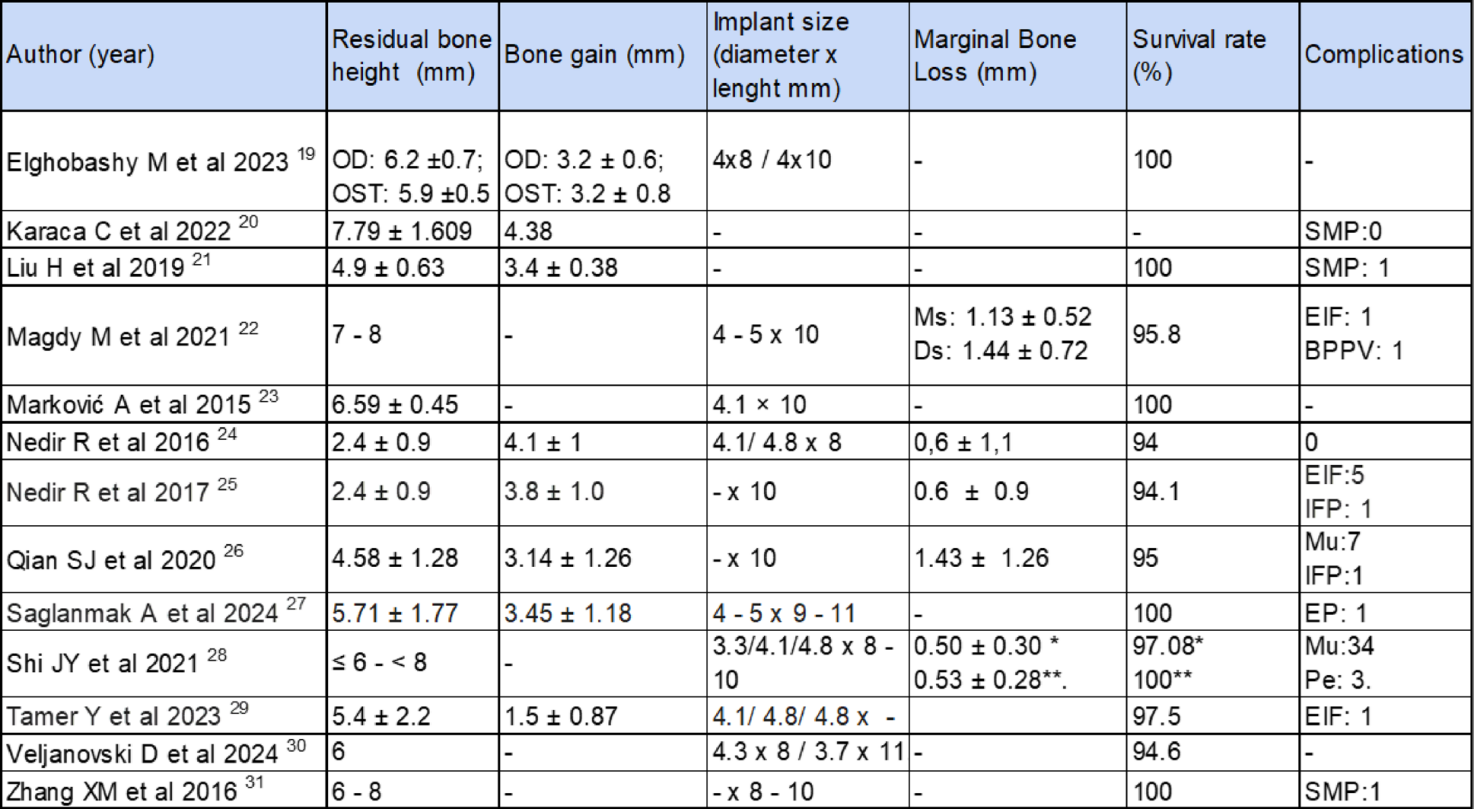
Results of individual studies

*OD* Osseodensification, *OST* Osteotome, *Ms* Mesial, *Ds* Distal, *SMP* Sinus Membrane Perforation, *EIF* Early Implant Failure, *IFP* Implant Failure due Peri-implatitis, *EP* Epistaxis, *BPPV* Benign Paroxysmal Positional Vertigo, *Mu* Mucositis, *Pe* Periimplantitis, * Group 1: 8 mm implants; ** Group 2: 10 mm implants

### Survival rate

Implant survival rates ranged from 94.1% to 100% using osteotomes and osseodensification techniques. [19, 21–31]

**Fig. 2:**
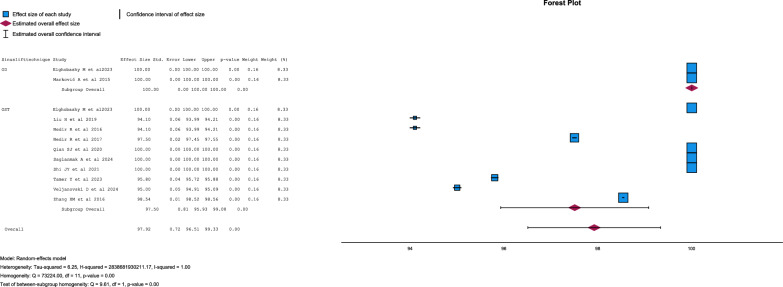
Forest-plot for implant survival

Implant survival for OD and OST was 100% [19, 27] and 97.5 ± 2.42% [19, 21, 26, 28, 29, 31] respectively with differences statistically significant (p = 0.002) (Fig. 2).

### Residual bone height

Four studies do not specify the mean for the RBH. Although, most studies describe that the RBH ranges from 6 to 8 mm [22, 28, 30, 31]^.^

The mean of RBH for OD technique was 5.95 ± 0.245 mm. With a minimum value of 5.71 mm and maximum of 6.2 mm [19, 30].

On the other hand, the mean RBH for OST technique was 4.995 ± 1.767 mm. The minimum value is 2.4 mm, and the maximum value is 7.79 mm [19, 21, 23, 26, 29]^.^

**Fig. 3:**
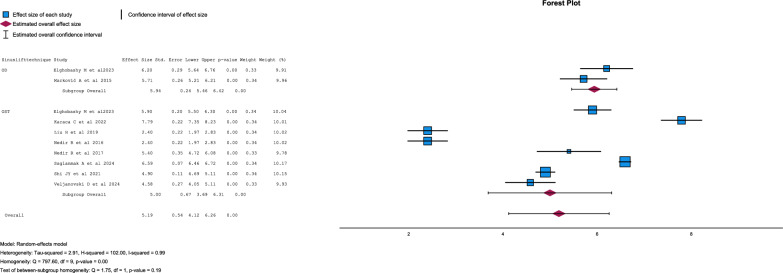
Forest-plot for residual bone height

**Fig. 4:**
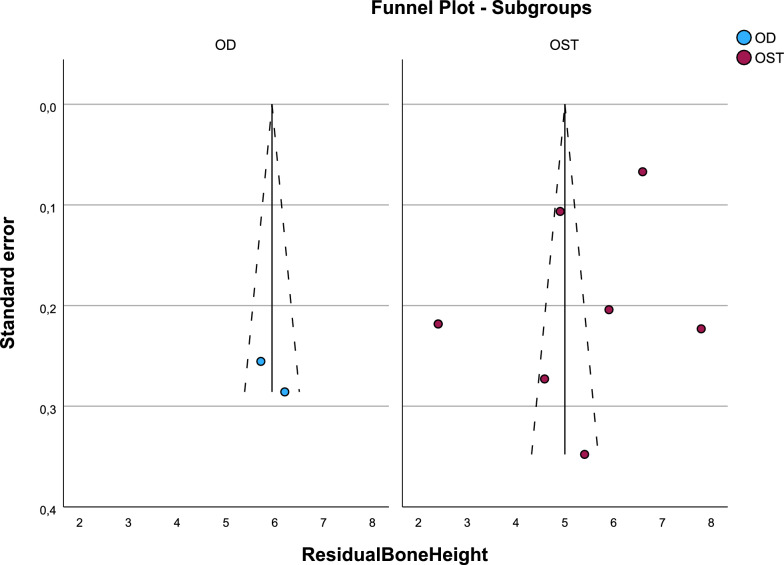
Funnel-plot for residual bone height

Residual bone height showed no statistical differences between both techniques (p = 0.186) (Figs. 3 and 4).

### Bone gain

Five studies do not register bone gain [22, 23, 28, 30, 31]. Regarding the OD technique, the average bone gain was 3.325 ± 3.325 mm. With a minimum value of 3.2 mm and maximum of 3.45 mm [19, 27]. In the OST technique group, the mean bone gain was 3.202 ± 3.2 mm. With a minimum value of 1.5 mm and maximum of 4.38 mm [19, 21, 24, 26, 29].

**Fig. 5:**
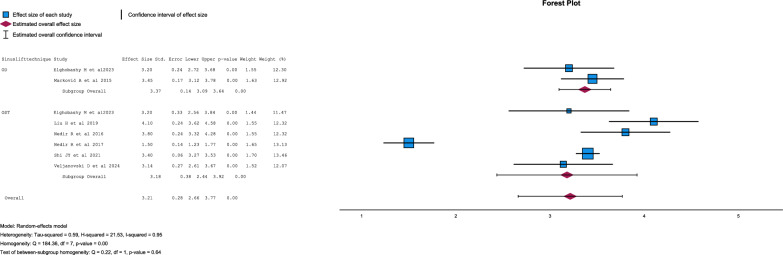
Forest-plot for bone gain

**Fig. 6:**
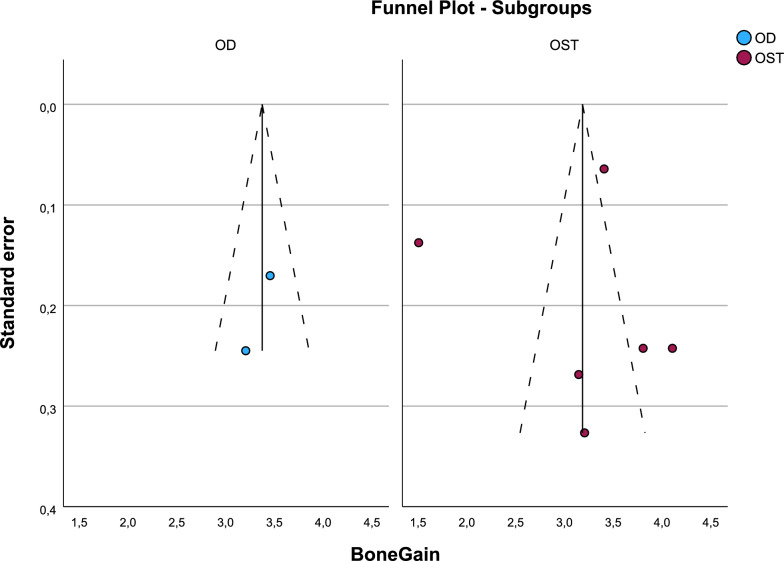
Funnel-plot for bone gain

There were no statistical differences between groups in bone gain (p = 0.639) (Fig. 5 and 6).

### Implant size

In the OD technique, the diameter of the implants described ranges between 3.7 and 5 mm [19, 27, 30]. In the OST technique, the diameter of the implants ranged between 3.3 and 5 mm [19, 22–25, 28–30].

Regarding the length of the implants placed, in both groups studied, implants ranging from 8 mm to 11.5 mm in length were placed [19, 22, 31].

For both groups, the most used diameter and length of the implant was 4 and 10 mm respectively.

### Complications

The most common complication in the OD technique was epistaxis, occurring in one out of 36 patients (2,78%) [27]. Regarding the OST technique, there were 2 cases of sinus membrane perforation among 103 patients (1,94%) [20, 21, 31], early implant failure occurred in seven of 72 patients (9,72%) [22, 25, 29], implant failed due peri-implantitis was observed in 2 cases of 34 patients (5,88%) [25, 26, 28], benign paroxysmal positional vertigo was presented on 1 cases of 24 patients (4,17%) [22] and mucositis was described in 41 cases of 154 patients (26,62%) [26, 28]; within the included studies, these complications are not reported by all the articles.

### Methodological quality and risk of bias of individual studies (Table 3 and 4)

**Table 3:**
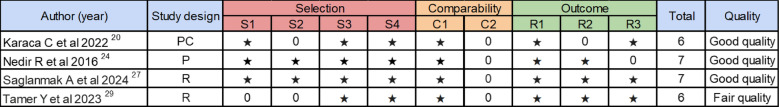
Newcastle–Ottawa Scale adapted for cohort and observational studies

Following an assessment of the cohort and observational study's methodological quality using the Newcastle–Ottawa Scale [17], it was concluded that the studies [20, 24, 27, 29] were good quality/ fair quality and presented a low risk of bias (Table 3). The Cochrane Collaboration identified RCTs that demonstrated a low risk of bias in six of the included studies [21, 23, 26, 28, 31], while three of them [19, 24, 30] presented unclear risk of bias (Table 4).

**Table 4:**
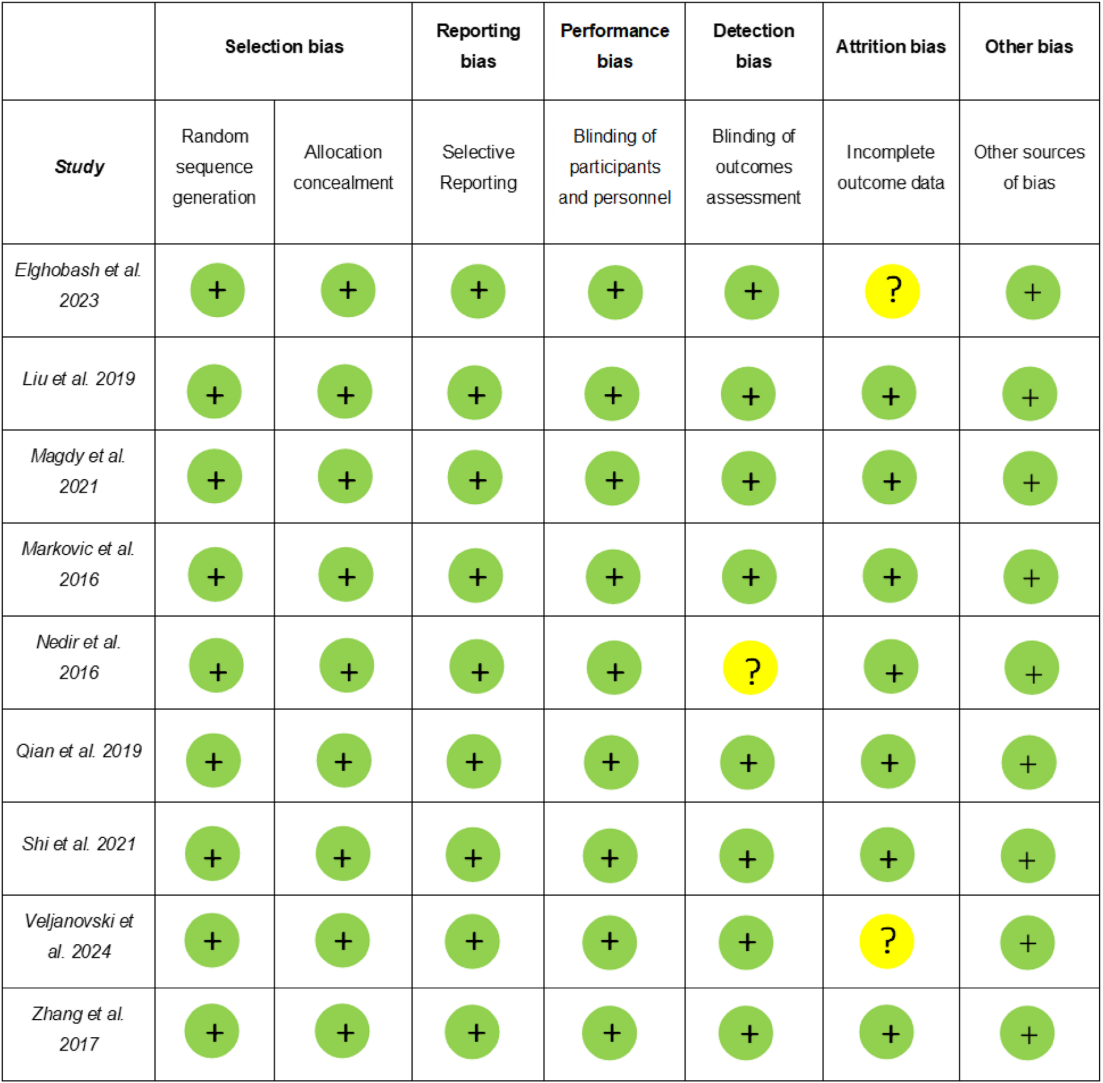
The Cochrane Collaboration’s tool for assessing risk of bias in randomised trials

## Discussion

### Summary of evidence

The aim of this study was to compare the outcomes of the classic crestal sinus lift technique and the osseodensification sinus lift approach in terms of the residual bone height, bone gain, implant size, implant loading, marginal bone loss, survival rate, follow-up time in addition to intra- and post-operative complications. The RBH did not show statistically significant differences between the two groups studied, with an average of 5.95 ± 0.245 mm for the OD group and 4.995 ± 1.767 mm for the OST group (p = 0.214), this allows bone gain to be analyzed from the context of starting from similar situations, as can be seen in the following section. As described above, the most commonly used diameter and length of the implant was 4 and 10 mm respectively for both groups, Potdukhe et al. [13], analyzes the stability of the implant in the OD and OST procedures, but does not specify the characteristics of the implants or their length, this value being important since it allows evaluating whether the bone gain was sufficient to place implants of conventional length. In our study we were able to confirm that sinus lift procedures with OD or OST are suitable for placing implants of at least 8 mm in length with simultaneous implant placement. In the systematic review and meta-analysis by Poonia and Patel [14], it was established that the osseodensification drilling protocol is superior to the use of osteotomes in patients with low bone density, resulting in better primary and secondary implant stability. While implant stability is a crucial factor for both survival and treatment success, our study focuses on other variables that provide a more comprehensive perspective and complement the data presented by these authors.

Decker et al. [15], conducted a review comparing the two techniques analyzed in this study. They noted that the OST technique has limitations, including patient discomfort during the procedure and the risk of vertigo, while regarding the OD technique, the limitations are still being studied, although they reported zero implant failures in the studies they included. In this study we analyze the complications associated with both techniques.

In all cases included in this review, the loading of implants was deferred, spanning a period of 10 weeks to 12 months. There are authors [30] who affirm in their studies that an advantage of the OD over the OST approach is that it has greater primary stability and therefore there are more possibilities of being able to carry out immediate loading. Among the variables to be evaluated in this study was MBL, however, we did not find studies that carried out the OD approach and evaluated this variable and it was only possible to report the results for the OST procedure. The results of both procedures indicate a high survival rate of the implants, which varies in a range from 94.1% to 100%.

### Bone gain

Regarding bone gain, there were similarity of outcomes between the groups studied in a follow-up period that ranged from 4 months to 10 years. Therefore, this systematic review determined that the results in regards to bone gain height in the classic crestal sinus lift technique are similar to those obtained with the osseodensification approach. However, it is important to note that only two studies in which the osseodensification approach was performed reported the results of bone gain. Therefore, the similarity of results of the two analyzed techniques may be due to the heterogeneity between the studies, small sample of patients and few studies included for this analysis. These findings corroborate the results obtained by Potdukhe, et al. [13], who reported a bone gain of 2.88 mm in the osseodensification approach and 2.57 mm of bone gain in the osteotome technique. Similarly, in the study by Decker et al., similar results were obtained to ours with respect to bone gain. For the OST group, the RBH was 5.7 mm and the final BH was 9.6 mm, with a bone gain of 3.9 mm, although data from only three studies were included. On the other hand, the results of the OD group differ from ours, since they report a bone gain of 6.05 mm, this may be because they include graft procedures. [15]

Furthermore, in almost all included studies (except for two authors [20, 21]), for both techniques it was possible to place implants of at least 8 mm in length. This is important, since it shows that the amount of bone gained was sufficient for implant placement in both scenarios.

Mester et al. [32] performed a systematic review and meta-analysis in which they concluded that maxillary sinus lift with lateral approach and simultaneous placement of conventional implants has a higher survival rate than the placement of short implants. On the other hand, Farina et al. [33] report in their RCT that transcrestal sinus lift has less postoperative morbidity and a more tolerable postoperative period for patients. In other words, with all the findings of this review and the data provided by previous studies, it can be said that transcrestal sinus lift with OD or OST approach allows simultaneous placement of conventional implants, which have a higher survival rate, with a lower postoperative morbidity and better postoperative period for the patient.

### Complications

Among the procedures assessed, OST technique reflected the complication rate 14.32%, compared to the OD technique, which showed a complication rate of 2.78%. This finding was in agreement with Potdukhe S.S et al [13], who concluded that osseodensificaton technique proved to have reduced morbidity and better postoperative outcomes. Similarly, the results concluded that no clinical membrane perforations or complications were observed during sinus lift surgery or postoperatively, throughout the 6-month follow-up period, in light of the fact that this study incorporates grafting [34]. According to Shalash M. et al. [35], in all the remaining cases in their study, where an allograft was utilized, no sinus perforation was detected clinically or radiographically. In contrast, in our study, graft procedures were not included.

The included studies utilizing the osteotome technique identified mucositis as the most frequent complication (26.62%), whereas other complications, such as sinus membrane perforation, were reported less frequently (1.94%), within the included studies, these complications are not reported by all the articles. However, these findings differ from the results of a systematic review by Ye M et al. [36], which identified sinus membrane perforation as the most common complication, accounting for 3.08% of total implants. Postoperative nosebleeds, paroxysmal vertigo, and infections were observed in only a small number of studies [36]. Similarly, the most frequently reported complication by Boyacıgil D.U et al. [37], during sinus lifting procedures is membrane perforation (18,2%), considering that it is a study employing grafting. In contrast to the study by Pozzi A. et al., [38], which used xenograft, no biological or mechanical complications including mobility, pain, or discomfort, were detected during the entire follow-up period.

Mester et al. [32] conducted a systematic review and meta-analysis, concluding that the biological complications (mucositis and peri-implantitis) differed between the group studying standard implants with sinus elevation and the group with short implants. However, no differences were observed in prosthetic complications between the two groups described. In contrast, Farina et al. [33], in their randomized controlled trial (RCT), indicated that the incidence of membrane perforation showed no differences between transcrestal and lateral approach. Interestingly, the higher incidence of nasal discharge/bleeding in the lateral group may be attributed to the increased (although not significantly) occurrence of membrane perforation. Moreover, orbital and periorbital subcutaneous emphysema (OPE) has also been described in association with the lateral approach. On the other hand, transcrestal approach was associated with a significantly lower incidence of swelling, bruising, and nasal discharge/bleeding compared to lateral approach. [33]

### Limitations and additional proposals

The main limitation of this meta-analysis was the limited number of RCTs, along with the variability in follow-up periods. Information was limited in some studies that do not describe the survival, the amount of bone gain and the residual bone height. Although, a few studies do not share detailed information for these variables which caused them to be excluded from the meta-analysis. Hence, only two studies describing OD have been used for meta-analysis.

Several studies did not clearly define the groups using grafts and those not using them, leading to confusion regarding implant survival, the amount of bone gain, implant placement, and complications. As aforementioned, there are not enough studies to describe intra- and post-operative complications specifically. The findings showed a greater inclination towards mucositis, with a higher percentage; however, this may be attributable to differences in sample size. Likewise, several studies have documented a relatively high incidence of sinus membrane perforations associated with osteotomes sinus floor elevation. [39]

Future studies could benefit from providing detailed data on the complications that arise, including their onset, progression, resolution, and prevention. In this regard, it will be essential to avoid factors that are related to complications occurring in sinus lift procedures—both when using the OD technique and the OST technique—by employing a more advanced statistical model and analyzing data from a more homogeneous set of studies, both clinically and methodologically, to achieve the most precise results and develop protocols that minimize risk.

## Conclusions

Considering the limitations in the present meta-analysis, it can be concluded that implant survival in OD technique is greater than in OST technique. There are no differences between OD and OST technique in terms of residual bone height and bone gain. OST technique describes higher rate of complications than OD technique. However, both techniques are safe, predictable and successful for implant placement.

More research should collect these data to allow for comparisons between different procedures, thereby generating complete information to aid clinicians in diverse clinical situations.

## Data Availability

No datasets were generated or analysed during the current study.
